# Moisture Absorption and Mechanical Degradation of Polymer Systems Incorporated with Layered Double Hydroxide Particles

**DOI:** 10.3390/polym16233388

**Published:** 2024-11-30

**Authors:** Stanislav Stankevich, Daiva Zeleniakiene, Jevgenijs Sevcenko, Olga Bulderberga, Katerina Zetkova, Joao Tedim, Andrey Aniskevich

**Affiliations:** 1 Institute for Mechanics of Materials, University of Latvia, Jelgavas St. 3, LV-1004 Riga, Latvia; 2 Department of Mechanical Engineering, Kaunas University of Technology, Studentų St. 56, 51424 Kaunas, Lithuania; 3 SYNPO a.s., S. K. Neumanna 1316, 532 07 Pardubice, Czech Republic; 4 DEMaC-Aveiro Institute of Materials, Campus Universitário de Santiago, 3810-163 Aveiro, Portugal

**Keywords:** moisture absorption, moisture concentration field, multilayered structure, layered double hydroxide, mechanical degradation, epoxy resin

## Abstract

This study investigated the moisture absorption and mechanical degradation of epoxy-based polymer systems with Mg-Al/NO_3_ layered double hydroxide (LDH) nanoparticles content up to 5 wt%. Such systems are developed for multilayer corrosion protective coatings. A sorption model was developed to calculate the moisture concentration field in the multilayer structures using Fick’s law of diffusion. The finite-difference method was used for the numerical solution. Epoxy/LDH nanocomposites were prepared using various dispersion methods with solvents, wetting agents, and via a three-roll mill. Moisture absorption was measured under different environmental conditions, including temperatures up to 50 °C and salinity levels up to 26.3 wt% salt solution. The results showed that equilibrium moisture content increased by 50% in hot water, while it was reduced by up to two times in salt solution. The diffusion coefficient in hot water increased up to four times compared to room temperature. The numerical algorithm was validated against experimental data, accurately predicting moisture distribution over time in complex polymer systems. Mechanical tests revealed that the elastic modulus did not change after water exposure; however, the ultimate strength decreased by 10–15%, especially in specimens with 5 wt% LDH.

## 1. Introduction

To extend the service life and reduce the maintenance costs of materials, particularly in aeronautical and offshore sectors, the use of protective coatings has become a widespread approach [1]. These coatings act as barriers against corrosive species in the environment, thereby protecting the underlying metal [2]. However, over time, these coatings can degrade due to natural ageing and external factors such as UV radiation [3], temperature fluctuations [4,5], mechanical stresses [6], and exposure to aggressive chemicals [7], leading to the formation of micropores and microcracks. These imperfections can compromise the coating’s integrity and allow for corrosion of the protected metal surface [8].

The application of inorganic, hybrid, or organic coatings, either in single- or multilayer forms, has been a common method for providing passive protection against corrosion. However, these systems are not without their challenges. Traditional corrosion inhibitors, such as those based on Cr(VI), have been used to provide active protection. Despite their effectiveness, Cr(VI) compounds are toxic and carcinogenic, prompting a significant research effort to find safer alternatives that offer similar levels of protection without the associated health and environmental risks [9]. The transition to environmentally friendly anti-corrosion additives is complex. Many alternative compounds, while effective in solution, do not perform as well in coating formulations due to adverse interactions with the coating matrix. Layered double hydroxides (LDHs), such as eco-friendly Mg-Al/NO_3_ LDHs, represent a promising class of nanomaterials [10,11,12]. These materials exhibit ion-exchange capabilities and anion adsorption properties. While the LDH particles themselves are not inherently anticorrosive [13], they have the potential to become so if modified with suitable anticorrosive agents within their layered structure, for instance, Ethylenediamine-N,N′-disuccinic acid [14], 8-Hydroxyquinoline [15], etc. Here, the focus is on understanding how LDH particles, when integrated into the polymer matrix, influence the fundamental properties of the polymer itself, such as its mechanical stability and moisture resistance. This foundational understanding of LDH–polymer interactions will provide a basis for future studies that aim to load the LDH particles with targeted materials to achieve desired functional outcomes, such as enhanced anticorrosive capabilities.

The absorption of moisture in polymer coatings, particularly multilayer systems enhanced with nanofillers, is a critical concern. Prolonged exposure to aqueous environments, such as saltwater, can significantly alter the mechanical properties and longevity of these materials [16,17], leading to structural degradation and increased maintenance costs. Current challenges in the field include the accurate prediction of moisture uptake and the subsequent mechanical property changes in these complex systems. A superficial description can often lead to inaccurate conclusions and frequently fails to account for the interactions between different layers, especially in systems modified with nanofillers, which can affect both the diffusion rates and the distribution of absorbed moisture.

The aim of this study is to characterise and model the moisture absorption of multilayer epoxy-based polymer structures filled with Mg-Al/NO_3_ LDH nanoparticles and to assess the mechanical degradation of the material after prolonged water exposure. By developing a comprehensive model for moisture absorption in these complex systems, this research serves to provide valuable insights into the design of more effective protective coatings. Such advancements will help optimise material performance, reduce maintenance costs, and extend the service life of critical infrastructure. The study’s focus will also be on creating a tool for moisture concentration calculation in multilayered structures, which could help to achieve more effective parameters of multilayered polymer coatings.

## 2. Analytical and Numerical Solutions of the Sorption Process in Single- and Multilayered Plates

### 2.1. Absorption Process in Monolayer Plates

Moisture sorption by homogeneous polymers, if it is governed by diffusion only, is traditionally described by Fick’s laws. Intentionally, we do not discuss the non-Fickian behaviour often observed in long-term sorption experiments, especially at elevated temperatures, as examined in other papers, e.g., [18], since it is outside this paper’s topic.

**Figure 1:**
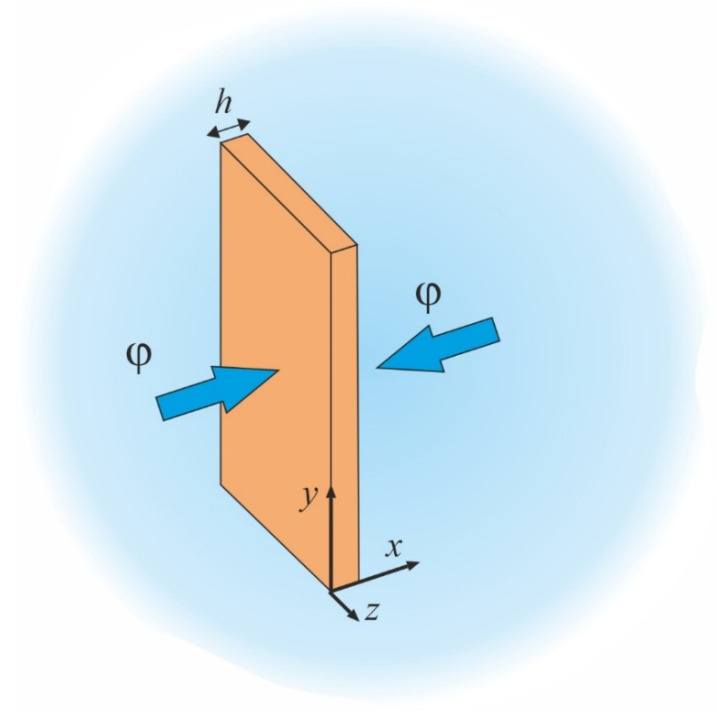
A thin plate absorbs moisture along the *x*-axis in a humid environment.

Consider 1D moisture diffusion along the *x*-axis in a thin plate of isotropic and homogeneous polymer in Cartesian coordinates [19,20]; see Figure 1. The specimen’s width and length are an order of magnitude greater than its thickness *h*. The relative humidity of the surrounding atmosphere is *φ*.

The changes in moisture concentration *c* with time *t* can be obtained from the partial differential equation:

(1) $$\frac{\partial c}{\partial t}=\frac{\partial }{\partial x}\left(D\frac{\partial c}{\partial x}\right)$$

where *D* is the diffusion coefficient that characterises the rate of moisture sorption.

Let us assume that *D* is independent of the moisture concentration. The solution of (1) can be more robustly obtained via the separation of variables method [21]. For initial $c\left(0<x<h,t=0\right)=c_{0}$ and stationary boundary $c\left(x=0,x=h,t>0\right)=c_{\infty }$ conditions, the solution is given the form of the series:

(2) $$c(x,t)=c_{\infty }-2\frac{\left(c_{\infty }-c_{0}\right)}{\pi }\sum_{k=1}^{\infty }\frac{\left(1-{\left(-1\right)}^{k}\right)}{k}\sin\left(\lambda_{k}x\right)\exp\left(-{\lambda_{k}}^{2}Dt\right),$$

where $\lambda_{k}=\pi k/h$. By integrating (2) across the plate thickness, the moisture content of the specimen can be obtained

(3) $$w(t)=w_{\infty }-2\frac{\left(w_{\infty }-w_{0}\right)}{\pi^{2}}\sum_{k=1}^{\infty }\frac{{\left(1-{\left(-1\right)}^{k}\right)}^{2}}{k^{2}}\exp\left(-{\lambda_{k}}^{2}Dt\right),$$

where $w_{0}$ and $w_{\infty }$ are the initial and equilibrium moisture contents of the specimen, respectively.

### 2.2. Calculation of the Moisture Concentration Field in Multilayered Structures

#### 2.2.1. Mathematical Model of Sorption

**Figure 2:**
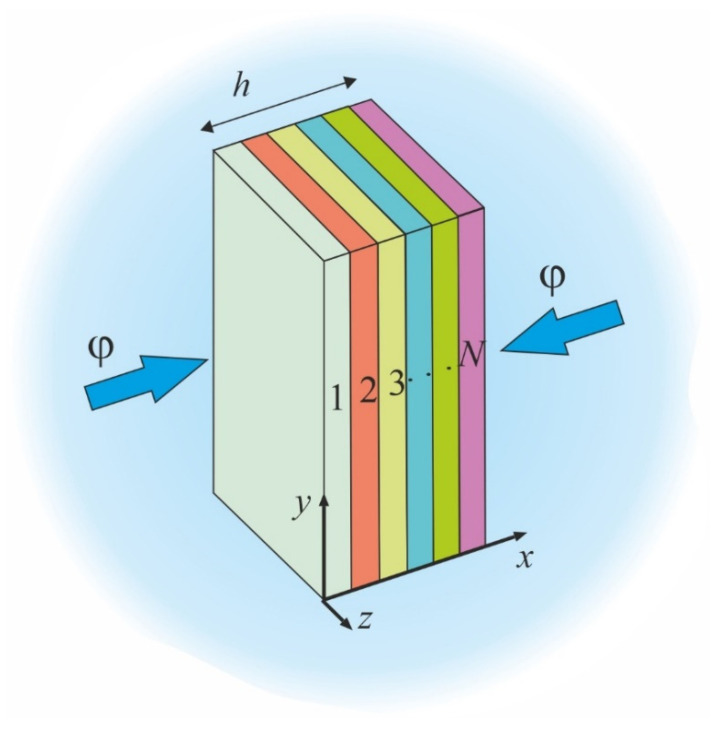
Scheme of the multilayer plate in an environment with relative humidity *φ*.

The moisture absorption in multilayered polymer structures often follows the same Fick’s law [22]. Let us examine the unidimensional sorption of moisture by a plane-parallel multilayer plate. We will assume that the plate consists of *N* layers, as shown in Figure 2. Diffusion of the moisture takes place along the *x*-axis. The plate is infinite in the other two directions. The layers have a clear interface. The unidimensional Fick’s equation describes the kinetics of diffusion in each layer

(4) $$\frac{\partial c^{i}}{\partial t}=D^{i}\frac{\partial^{2}c^{i}}{\partial x^{2}};i=1,\ldots ,N,$$

where *D^i^* is the moisture diffusion coefficient, and *c^i^* is the moisture concentration in the *i*-layer.

The layers may be anisotropic in the general case, but we assume that the *x*-axis coincides with one of the principal axes of the diffusion coefficient tensor.

The relative moisture content *W* at the current time moment *t* is the specimen’s volume-integrated moisture concentration and is experimentally determined as the relative increase in the mass of the specimen due to the absorption of moisture:

(5) $$W(t)=\frac{p(t)-p_{0}}{p_{0}}\cdot 100\%,$$

where *p* and *p*_0_ are the current and initial mass of the specimen, respectively. Along with the diffusion coefficient, a sorption isotherm is assigned for each layer—the dependence of the equilibrium moisture content $W_{\infty }^{i}$ of the *i*-layer on the relative humidity of the environment *φ*.

(6) $$W_{\infty }^{i}=f^{i}(\varphi ),$$

where *f^i^* is an isotherm empirical function whose analytic form in the general case is different for each layer and is chosen based on the best approximation of the experimental data. The linear form of this dependence is well known as Henry’s law. Nonlinear dependencies are known as Brunauer, Emmett, and Teller (BET) classification, but we do not discuss isotherm classification in this paper. The following expression is often used to describe isotherms phenomenologically.

(7) $$W_{\infty }^{i}=a^{i}\varphi^{b^{i}};\;a^{i},b^{i}=const.$$

Constant values of relative moisture content for the medium are assigned to the external boundaries of the plate and correspond to the relative humidity of the environment. The temperature is assumed to be constant over time and uniform across the plate.

#### 2.2.2. Transport of Moisture at the Interface of the Layers

**Figure 3:**
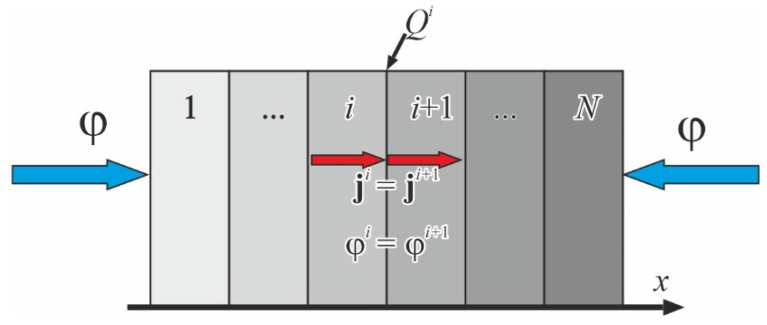
The interface between two layers has equality of the diffusional flows (8) and moisture transport potentials (9) at the interface *Q^i^*. Red vectors of the diffusional flows are directed along the *x*-axis only for clarity.

Since the second-order equation describes moisture transport (4), two conditions must be imposed on concentration and its derivatives with respect to the coordinates to ensure the uniqueness of the solution at the interface of the layers *i* and *i* + 1. This interface is designated as *Q^i^*, with an ideal contact between the layers as in Figure 3.

The first condition is the equality of vectors of the diffusional flows **j** that measure the amount of water that flows through a unit area during a unit time interval. This condition follows from the mass conservation law:

(8) $$|j^{i}|=|j^{i+1}|.$$

As the second condition, we take the equality of the chemical potentials $\mu^{i}=\mu^{i+1}$ following the laws of thermodynamics. The chemical potential is expressed in this case as μ=RTlnφ. In the absence of temperature gradients, the second equation can be regarded as the equality of the moisture transport potentials [23,24]

(9) $$\varphi^{i}=\varphi^{i+1}.$$

In the case of diffusional absorption, the moisture transport potential for an elementary volume *dv* is numerically equal to the relative humidity of the medium at which the volume is in sorption equilibrium. We assume that the moisture distribution is uniform within the volume of *dv*. That means we have identical values of moisture transport potential *φ*^I^ and *φ*^II^ for specimens of two different materials that are in equilibrium with the environment. Moreover, these potential values are numerically equal to the relative humidity of the medium, *φ*^I^ = *φ*^II^ = *φ*. If these two specimens are joined to form a two-layer plate, the existing equilibrium still occurs, and no moisture is transported across the interface. Thus, the potential remains constant at all points of the plate. The relative moisture contents of the layers *W*^I^ and *W*^II^ are different and determined by (6).

If the multilayer plate is not in equilibrium, the dependence of potential on the coordinates is still a continuous and smooth function. The moisture concentration at the interface undergoes a discontinuity. The jump value depends on the properties of the layers’ materials and the current moisture content. This jump shows the principal difference between moisture transport and heat conduction in a multilayer plate, since there is no temperature jump at the interface.

Taking into account (6), we can rewrite the condition (9) in the following form:

(10) $${\left[f^{i}(w^{i})\right]}^{-1}={\left[f^{i+1}(w^{i+1})\right]}^{-1},$$

where $[f^{i}(w^{i}){]}^{-1}$ and $[f^{i+1}(w^{i+1}){]}^{-1}$ are the inverses of $f^{i}(\varphi )$ and $f^{i+1}(\varphi )$, respectively. The analytic form $f^{i}(\varphi )$ may differ for different layers. At least in case (7), coefficients *a* and *b* are different for various layers. Thus, in the general case (10), there is no analytic solution. As a result, neither solves the problem as a whole.

#### 2.2.3. Algorithm for Numerical Solution of the Problem

To solve the abovementioned problem, we resorted to the finite-difference method using a difference scheme [25]. Following this approach, the derivatives of functions are replaced by their finite-difference analogues for each layer:

(11) $$\frac{\partial c}{\partial t}\Rightarrow \frac{c_{j,k+1}-c_{j,k}}{\Delta t},$$

(12) $$\frac{\partial^{2}c}{\partial x^{2}}\Rightarrow \frac{1}{\Delta x}\left(\frac{c_{j-1,k}-c_{j,k}}{\Delta x}-\frac{c_{j,k}-c_{j+1,k}}{\Delta x}\right)=\frac{c_{j-1,k}-2c_{j,k}+c_{j+1,k}}{\Delta x^{2}},$$

where the subscripts *j* and *k* denote changes in concentration along the coordinate and over time, respectively. The diffusion Equation (4) is replaced by its analogue

(13) $$\frac{c_{j,k+1}-c_{j,k}}{\Delta t}=D\frac{c_{j-1,k}-2c_{j,k}+c_{j+1,k}}{\Delta x^{2}}.$$

**Figure 4:**
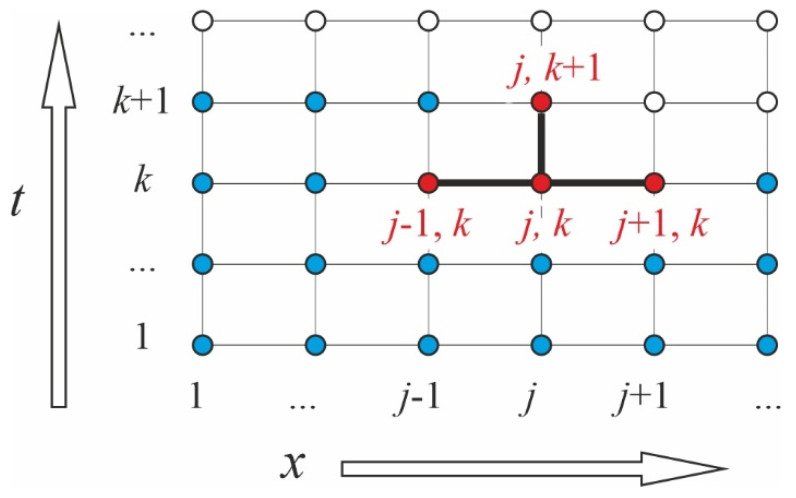
Grid pattern for the diffusion equation solved by the finite-difference method.

We assume that the initial distribution of moisture over the cross-section of each layer is uniform, but the concentration changes from layer to layer. We divide each layer by a grid with the mesh ∆*x^i^*. Thus, as the initial condition, we assign a concentration field at all grid points for the sandwich as a whole. We assign constant values of moisture concentration at the outer boundaries of the sandwich, corresponding to the relative humidity of the environment. Conditions (8) and (10) for each interface appear as follows when expressed in terms of finite differences:

(14) $$D^{i}\frac{c_{j,n-1}^{i}-c_{j,n}^{i}}{\Delta x^{i}}=D^{i+1}\frac{c_{j,1}^{i+1}-c_{j,2}^{i+1}}{\Delta x^{i+1}},\;i=1,\ldots ,N-1,$$

(15) $${\left[f^{i}\left(\frac{c^{i}}{\rho^{i}}\right)\right]}^{-1}={\left[f^{i+1}\left(\frac{c^{i+1}}{\rho^{i+1}}\right)\right]}^{-1},$$

where ρ*^i^* is the density of the *i*-layer. The problem’s solution comes down to a sequential search for moisture concentration values at each grid node throughout the sandwich. In the present case, the grid appears as in Figure 4. The red dots are involved in the current calculation loop. Blue dots are already calculated for the current moment, and white dots will be calculated.

We rearranged (13) to calculate the concentration at the moment *k* + 1

(16) $$c_{j,k+1}^{i}=\left(1-2\frac{\Delta tD^{i}}{{\left(\Delta x^{i}\right)}^{2}}\right)c_{j,k}^{i}+\frac{\Delta tD^{i}}{{\left(\Delta x^{i}\right)}^{2}}\left(c_{j-1,k}^{i}+c_{j+1,k}^{i}\right).$$

A necessary condition for calculation convergence is the inequality for each layer

(17) $$\Delta t\leq {\left(\Delta x^{i}\right)}^{2}/2D^{i},$$

which should be checked for each layer before the calculation starts, and the grid and time step should be adjusted if necessary. The accuracy and duration of the calculation are significantly affected by the ∆*t* and ∆*x^i^*.

#### 2.2.4. Interface Jump of the Concentration

**Figure 5:**
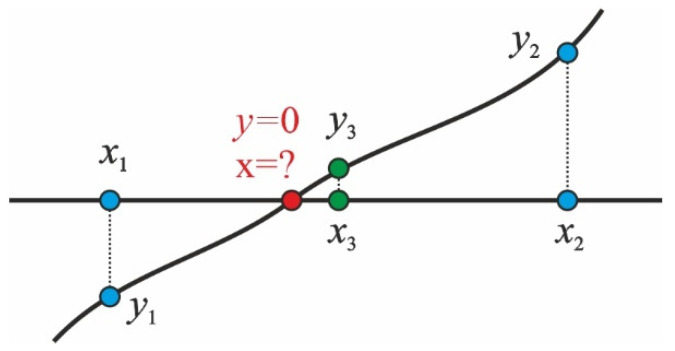
The bisection method is illustrated, with more comments in the text.

Equations (14) and (15) should be solved simultaneously to find concentration values from both sides of the interface *Q^i^* for all *N*-1 interfaces at each time step. The numerical solution was performed using the bisection method illustrated in Figure 5. Some smooth discontinuous function *y*(*x*) contains a root, and we must find the *x* value when *y* = 0, a red dot. Consider a line segment *x*_1_*x*_2_ such as *y*_1_·*y*_2_ < 0, all blue dots. We set a new point *x*_3_ in the middle of the segment *x*_1_*x*_2_ and find *y*_3_ value, green dots, which we compare with *y*_1_ and *y*_2_ values. Values *y*_3_ and *y*_2_ are the same sign, so the first iteration is performed, and the next choice is the segment *x*_1_*x*_3_.

Thus, after some iterations, we can find the root with a given in advance accuracy.

#### 2.2.5. Flowchart

**Figure 6:**
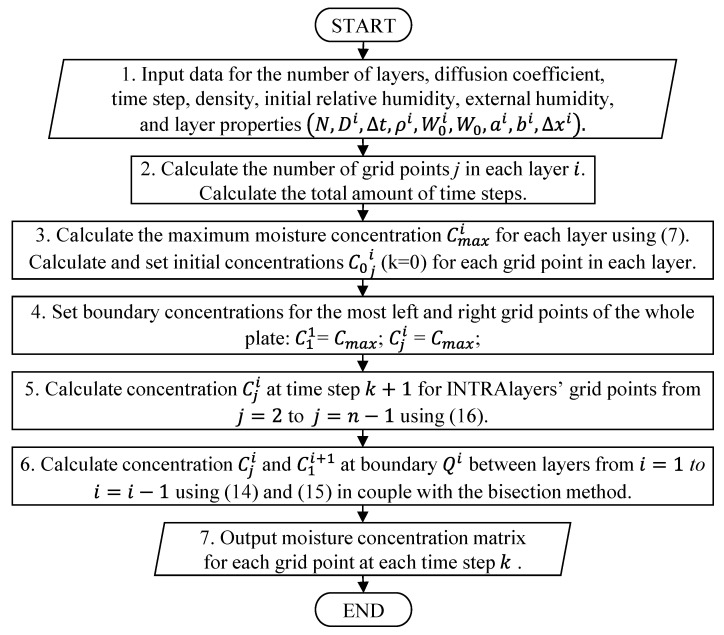
Flowchart for moisture concentration field calculation in polymer multilayered systems.

The primary computation process of moisture concentration field calculation has been consolidated into a flowchart, as shown in Figure 6. The flowchart illustrates the computational process for simulating the distribution of moisture concentration within a multilayered material over time. The procedure begins by inputting the necessary parameters (step 1), including the number of layers, material properties, environmental conditions like external humidity, and simulation parameters such as time steps and diffusion coefficients. These inputs define the moisture behaviour within each layer and across the whole material. Following data input, the model calculates the number of grid points for each layer and determines the total time steps required for the simulation (step 2). This discretisation process sets up the computational grid, allowing for the detailed tracking of moisture movement through the material. Next, the maximum potential moisture concentration for each layer is calculated based on the given conditions, and initial moisture concentrations are assigned to each grid point (step 3). This ensures that the simulation starts from a well-defined state. Boundary conditions are then applied to the outermost grid points of the material (step 4), setting their moisture levels according to the environmental humidity. The model can be adapted to different moisture concentration levels on the left and right sides of the material, allowing for more realistic simulations of environmental conditions. These boundary conditions play a crucial role in accurately representing the interaction between the material and its surroundings.

During the calculation procedure, all data can be stored in a temporary matrix for intermediate steps. This allows for efficient management and the retrieval of data during computations. The specifics of this step can vary significantly depending on user needs, including the amount of data stored and their organisation in the code. The temporary matrix can help streamline the process by facilitating access to intermediate results or by providing additional data for further analysis. The simulation proceeds with iterative calculations, where the moisture concentration is updated at each grid point inside the layers (step 5). For points located at the interfaces between adjacent layers (step 6), the bisection method that was described in Section 2.2.4 is used to ensure the continuity of moisture transfer across layers with differing properties. This ensures the physical accuracy of the model by addressing variations in material characteristics between layers. Finally, the results are output, providing a detailed concentration matrix that tracks the changes in moisture distribution across the entire material over time (step 7). This matrix enables a comprehensive analysis of moisture behaviour, revealing how moisture migrates through each layer and responds to external conditions. The flowchart is designed to be adaptable across different programming environments, making it a versatile tool for implementation in various computational settings. This adaptability ensures that the method can be tailored to the specific needs of different users and systems, making it suitable for a wide range of applications in material science, building physics, and other fields where moisture dynamics are critical.

## 3. Materials and Methodology

### 3.1. Compound Preparation

The integration of LDH into an epoxy matrix poses challenges, particularly in achieving uniform particle dispersion [26,27]. The need for surface functionalisation or the use of wetting agents to enhance particle compatibility with the polymer often arises. When the direct incorporation of LDH particles in powder form is impractical, dispersing the particles in a solvent can be a viable alternative. To address these challenges, three distinct approaches for particle integration were investigated: (1) using xylene as a dispersing solvent, (2) employing BYK 180 (supplied by BYK Additives & Instruments, Wesel, Germany) as a wetting agent, and (3) direct incorporation of LDH particles without intermediaries using 3-roll mill machine.

#### 3.1.1. Epoxy/LDH Nanocomposites with Xylene

**Figure 7:**
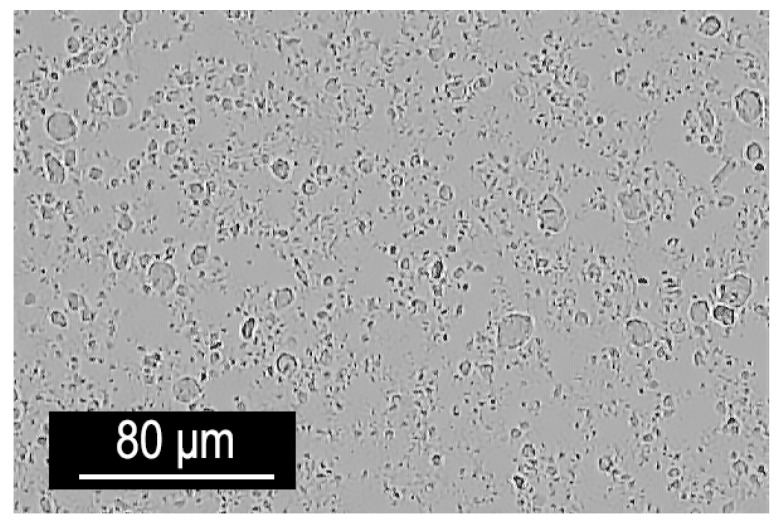
Optical micrograph of the epoxy mixture containing 2 wt% LDH incorporated through the xylene/LDH masterbatch.

The method utilised MgAl-NO_3_ LDH particles dispersed in a water-based slurry (wt% solids: 15–20), which was first processed through a nine-step washing procedure to replace the water with ethanol, acetone, and finally xylene. The slurry was mixed with ethanol in a 1:4 ratio and centrifuged at 6000 rpm for 15 min. This process was repeated three times. Subsequent washes with acetone and xylene followed, transforming the LDH suspension into a gel-like xylene solution containing 26 wt% LDH. This xylene-based LDH gel was added to the bisphenol A epoxy resin CHS 582 (supplied by Synpo a.s., Pardubice, Czech Republic). The mixture underwent high-shear mixing at 15,000 rpm for 5 min, followed by ultrasonic mixing to ensure thorough particle dispersion (Figure 7). The hardener Telalit 0420 (Synpo a.s., Pardubice, Czech Republic) was added in a 1:4 ratio to the epoxy, and the resulting mixture was manually stirred for 10 min before degassing under vacuum to eliminate air bubbles. This process yielded a uniformly dispersed LDH–epoxy nanocomposite.

As observed in the optical microscope image, complete delamination of the particles down to individual particles was not achieved. However, the overall distribution of particle agglomerates throughout the material volume was stable. Therefore, it can be concluded that the specimens produced from this mixture exhibit a uniform structure.

#### 3.1.2. Epoxy/LDH Nanocomposites with BYK

**Figure 8:**
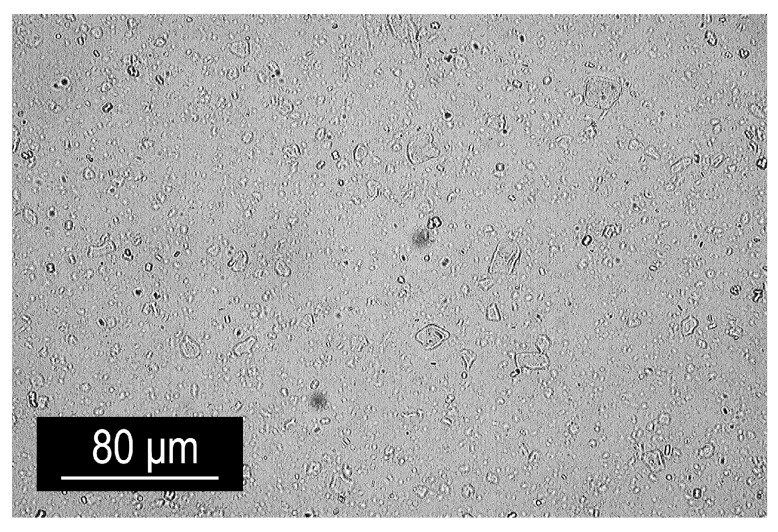
Optical micrograph of the epoxy mixture containing 2 wt% LDH incorporated with the use of BYK 180.

An alternative method for particle incorporation involved using MgAl-NO_3_ LDH particles in powder form with diameters of 5–10 μm, supplied by Smallmatek (Aveiro, Portugal). To achieve high-quality dispersion, the wetting additive Disperbyk BYK 180 was employed. BYK was added at a ratio of 1:2.3 relative to the LDH powder and was ground in a mortar for 15 min to achieve a fine texture. This paste was then blended with the hardener under 15,000 rpm for 15 min using a high-shear mixer. To ensure a uniform mixture, the blend was vacuumed for 30 min before adding the epoxy resin. The mixture was then stirred for another 10 min and subjected to a further 10 min of vacuuming to remove any residual air bubbles before finally being mixed with epoxy resin. Similarly to the previous preparation method, LDH agglomerates were observed (Figure 8).

However, despite the absence of a solvent, the overall distribution of particles within the volume was uniform and comparable in homogeneity to that achieved with the xylene method.

#### 3.1.3. Direct Incorporation Without BYK or Xylene

**Figure 9:**
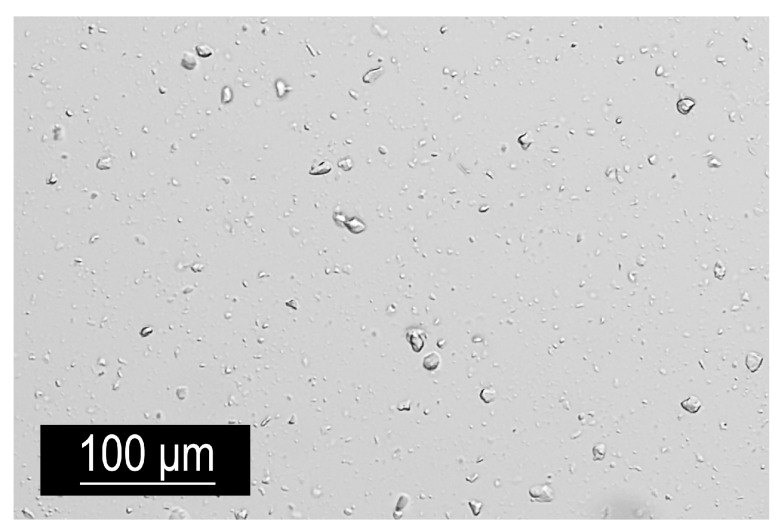
Optical micrograph of the epoxy mixture containing 2 wt% LDH prepared using three-roll mill machine.

The approach involved the direct incorporation of LDH particles in a powder form into the epoxy matrix without the use of additional agents. For this, a 6.2 wt% LDH-epoxy masterbatch was prepared using a three-roll mill. LDH particles were directly mixed into the epoxy resin. Approximately 80 g of the masterbatch were processed at a time, with 5 to 8 passes completed at each stage as the gap between the rollers was reduced from 60 to 40, 20, and finally 10 μm. This method ensured a uniform dispersion of LDH particles within the epoxy matrix. However, even considering the use of a slower and more complex masterbatch preparation process, the images (Figure 9) reveal the presence of agglomerates, similar to those observed in previous methods. This suggests that high-shear mixing is sufficiently effective at dispersing particles throughout the epoxy matrix. The prepared masterbatch was subsequently diluted with epoxy resin to achieve the desired particle concentration in the composition. All prepared unique compounds detailing the concentrations of each component, including LDH, epoxy resin, hardener, and any additional agents, are summarised in Table 1.

### 3.2. Specimens

To evaluate the mechanical properties and absorption characteristics of the epoxy nanocomposites, 24 specimens of each unique composition were fabricated. The only exception was the composition without xylene and BYK additives. Due to the time-intensive fabrication process and the reduced yield of the final masterbatch, specimens from this mixture were fabricated solely for analysing the degradation of mechanical properties under moisture exposure. Rectangular bars for tensile tests were fabricated with dimensions of 100 × 10 × 1.5–2 mm. Due to the simple geometry, the same bars were utilised for absorption measurements as well. These specimens were produced from epoxy mixtures with varying LDH concentrations (0, 1, 2, and 5 wt%) using the previously described methods. After casting, the bars underwent a carefully controlled post-curing process, which involved three temperature steps: 50 °C for 2 h, 80 °C for 1 h, and 120 °C for 1 h. This post-curing temperature program ensured complete polymerisation and optimised the mechanical properties of the nanocomposites. Dog bone specimens, with a gauge section cross area of 3 × 1 mm conforming to the ISO 527-2 standard’s specimen Type 5A, were prepared to assess more precise tensile properties and to evaluate mechanical degradation. The preparation process for these specimens was analogous to that of the tensile bars, involving casting, post-curing following the same temperature program, and polishing.

### 3.3. Mechanical Testing

Rectangular bars and dog bones were utilised for mechanical tests. Tensile tests were performed using the universal testing machine Zwick 2.5k (Zwick Roell Group, Ulm, Germany), with a loading speed of 1 mm/min. For the more accurate and consistent testing of mechanical properties, a clip-on extensometer 5025 (supplied by Zwick) was used with a base length of 30 mm (resolution 0.1 μm, class 0.5 to EN ISO 9513).

### 3.4. Absorption Measurement Procedure

The absorption measurement procedure was performed under several environmental conditions, including variable temperature and salt content. The procedure involved systematically weighing and recording specimens over a specified period until equilibrium moisture content *w_∞_* was reached. Before immersion, the mass and geometric dimensions of all specimens were carefully measured to establish the reference values. The specimens were immersed in four distinct aqueous environments: distilled water (DW) at room temperature of 21 °C, distilled water at an elevated temperature of 50 °C, a 3.4 wt% Atlantic salt solution at room temperature [28], and a saturated salt solution of 26.3 wt% Atlantic salt at room temperature. A high temperature and salt concentration were selected to achieve the maximum possible impact on sorption parameters as well as on the mechanical properties of the matrix. Over the experiment, the mass of each specimen was periodically measured. The measurement intervals were set as follows: on the 1st, 2nd, 3rd, 5th, 7th, and 13th days, followed by weekly measurements thereafter. This schedule allowed for tracking the progression of moisture absorption over time. During each measurement, the specimens were briefly removed from their respective aqueous environments. Specimens immersed in salt solutions were quickly rinsed with distilled water to prevent salt residues from affecting the mass readings. Excess surface moisture was carefully blotted away using a lint-free paper towel, ensuring accurate weight measurement. The specimens were weighed using a precision analytical balance XS205 (supplied by Mettler Toledo, Columbus, OH, USA) with a minimal readability of 0.01 mg. To minimise errors due to drying, the weighing of all specimens was conducted in the same order during each measurement session, after which the specimens were promptly returned to their respective environments. The mass measurements obtained over time were used to calculate the amount of absorbed moisture at each time point *w_t_*, relative to the initial mass *w_0_*. The moisture content at the time *t* was calculated using (5). These data were used to construct sorption curves, which characterise the kinetics of moisture absorption. The curves plotted the moisture content as a function of the square root of time, allowing for the analysis of the absorption behaviour of the specimens and their degradation in different environments.

## 4. Characterisation

### 4.1. Sorption Parameters

**Figure 10:**
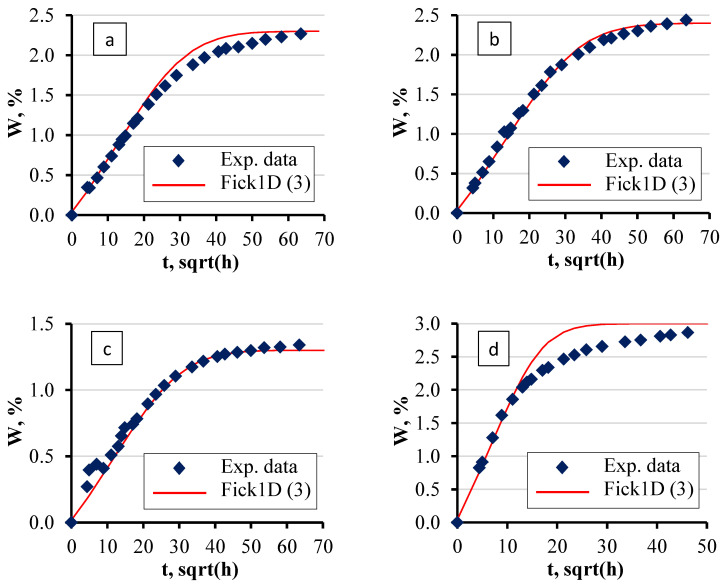
Representative sorption curves of neat epoxy + BYK specimens in four water solutions: (**a**) Atlantic water at 21 °C, (**b**) distilled water at 21 °C, and (**c**) saturated salt solution at 21 °C; (**d**) distilled water at 50 °C. The average deviation is less than the size of the experimental dots.

The sorption behaviour of the epoxy/LDH nanocomposites with BYK and xylene (see “B” and “X” in Table 1) was characterised by analysing the sorption curves and parameters obtained under different environmental conditions. As depicted in Figure 10, the sorption curves for specimens prepared using BYK demonstrate that the sorption parameters were significantly influenced by the surrounding environment. Notably, both the sorption rate and equilibrium moisture content varied depending on the medium in which the specimens were immersed. The highest equilibrium moisture content was observed in specimens exposed to hot water, where the values surpassed those in other conditions.

The experimental data were fitted using the analytical solution of the 1D Fick Equation (3). This allowed for the calculation of sorption curves across all specimen groups. The curves were constructed based on sorption parameters that were iteratively selected and refined during the experiments. As shown in the sorption curves, the specimens within each group exhibited good repeatability, allowing for the use of common sorption parameters for modelling purposes across each group. The rate of moisture absorption in specimens exposed to hot water was so high that the model struggled to accurately describe the sorption curve as precisely as it did for the cases with specimens in water solutions at room temperature. This discrepancy suggests that the higher diffusion rates at elevated temperatures introduce complexities that the model does not fully capture, particularly in the early stages of sorption.

**Figure 11:**
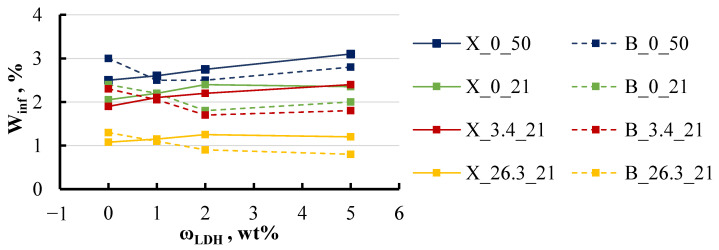
The equilibrium moisture content of xylene and BYK specimens (“B” and “X” in Table 1) with different LDH contents in various water solutions. Notations: X or B_salt content (wt%)_temperature (°C).

**Figure 12:**
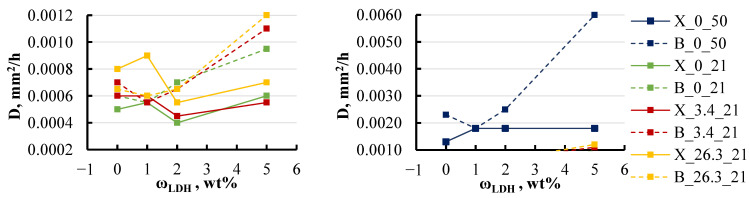
The diffusion coefficient of xylene and BYK specimens in various water solutions. Notations: X or B_salt content (wt%)_temperature (°C).

The sorption parameters, such as equilibrium moisture content and diffusion coefficient, were found to be dependent not only on the temperature of the medium but also on its salinity, as shown in Figure 11 and Figure 12.

The highest equilibrium moisture content was observed in specimens immersed in 50 °C water with no salt, with values that were three times higher than those of specimens stored in a saturated salt solution. The filler content in the specimens had a minimal impact on the equilibrium moisture content, and similarly, the influence of BYK and xylene in the specimens was also negligible.

The diffusion coefficient exhibited even less variation across the different conditions, with the notable exception of specimens stored in a hot environment. For these specimens, the diffusion coefficient increased by a factor of four and even more with the LDH content increase. Temperature emerged as a significant factor influencing the sorption parameters. As the temperature increased from 21 to 50 °C, both the diffusion coefficient and equilibrium moisture content rose across all specimen groups. Specifically, at 50 °C, the diffusion coefficient increased by 3–4 times for most groups, with the exception of the group containing the highest BYK content, where it rose by 6 times. Meanwhile, the equilibrium moisture content increased consistently by 30–40% across all groups, regardless of whether they contained BYK or xylene. This trend underscores the importance of temperature in determining the sorption behaviour of the epoxy/LDH nanocomposites, affecting both the rate of moisture diffusion and the overall moisture retention capacity.

**Figure 13:**
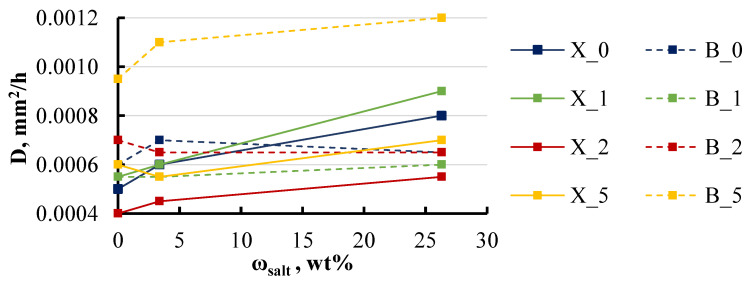
Effect of the solution’s salt concentration on the diffusion coefficient of xylene and BYK specimens. Notations: X or B_LDH content (wt%).

The presence of salt did not significantly affect the diffusion coefficient, as corroborated by the data presented in Figure 13.

For specimens with BYK, the presence of salt had no impact on the diffusion coefficient, with the exception of the group containing the highest BYK content of 5 wt%. In contrast, for specimens with xylene, an increase in salt concentration from 0 to 26 wt% led to a noticeable increase in the diffusion coefficient across all groups, ranging from 30 to 40%.

**Figure 14:**
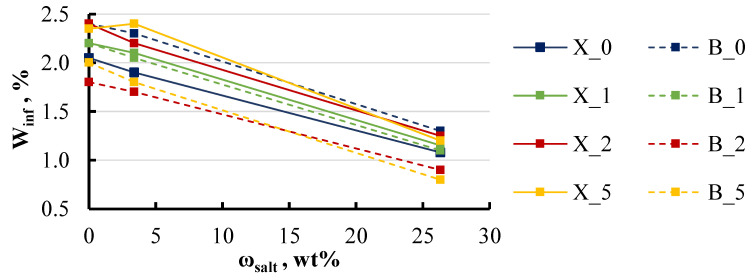
Effect of the solution’s salt concentration on the equilibrium moisture content of xylene and BYK specimens. Notations: X or B_LDH content (wt%).

However, the impact of salt on the equilibrium moisture content was more pronounced. Higher salt concentrations led to a marked decrease in equilibrium moisture content (Figure 14). As the salt concentration increased from 0 to 26 wt%, the equilibrium moisture content decreased by 50% for both the xylene and BYK specimens.

The acquired data show that the moisture characteristics of the surrounding environment play an important role in governing both the extent and dynamics of moisture penetration into the material.

### 4.2. Mechanical Tests and Degradation

**Figure 15:**
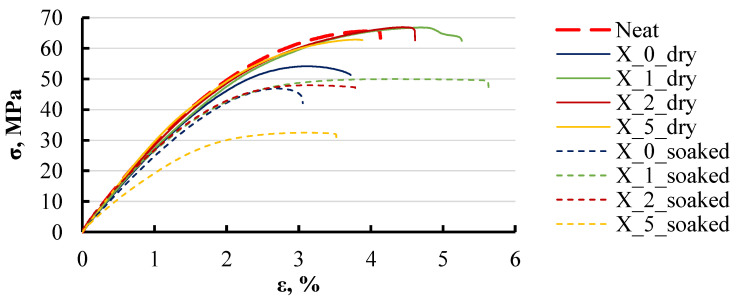
Representative stress–strain curves of xylene-based dry and soaked specimens in 3.4 wt% water solution. Notations: X_LDH content (wt%)_specimen state.

To assess the degradation of the mechanical properties of the nanocomposites under the influence of aqueous environments, tensile tests were conducted. The representative stress–strain curves for these tests are presented in Figure 15, which shows the behaviour of specimens with the addition of xylene. The curves include data for both dry and soaked specimens, clearly illustrating a decrease in material strength following water exposure.

The maximum stress for all curves corresponding to soaked specimens decreased by 10–50%, highlighting the impact of moisture on the mechanical properties of the material. The amount of xylene in the group with 0 wt% of LDH was matched to the quantity used in the 5 wt% LDH specimens, ensuring a comparable solvent content. The inclusion of xylene in the 0 wt% LDH formulation resulted in a slight decrease in strength of about 10–15%, potentially because it was the only instance where xylene was added in its pure liquid form rather than as part of a masterbatch.

**Figure 16:**
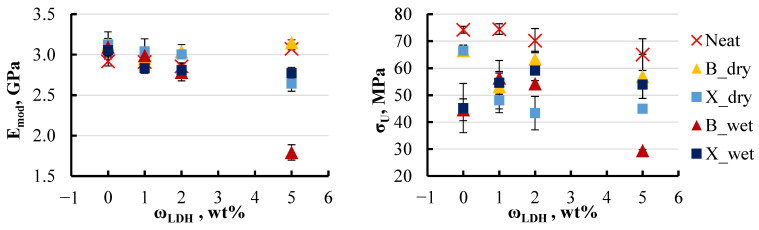
Effect of LDH content on elastic modulus (**left**) and ultimate strength (**right**) of dry and soaked (in 50 °C water) xylene and BYK specimens.

The initial focus was on determining how soaking affects the mechanical properties of the material. To observe the most pronounced changes, the most aggressive environment of hot water was chosen, as it leads to significant moisture absorption. Figure 16 provides a comparison between dry specimens and those soaked in hot water.

The results indicate that the elastic modulus changes only slightly, even after prolonged exposure to hot water. The decrease in elastic modulus is around 5–8% with the addition of LDH up to 2 wt%. For specimens with xylene, the modulus continued to decrease even with the higher filler content, resulting in an overall reduction of 10% from the initial modulus. The trend changes when the LDH content is increased to 5 wt% for BYK and neat specimens. Dry specimens, both without additives and those with BYK, showed a return to their initial modulus. In contrast, wet specimens with BYK experienced a significant drop in modulus, decreasing by up to 45%. Such a significant decrease in the modulus observed in wet specimens with BYK may be attributed to the fact that BYK is a wetting agent. As shown by previous results, this characteristic of BYK substantially alters the sorption properties of the material, drawing more moisture into the specimens. The impact is more dynamic when examining ultimate strength. A significant drop in ultimate strength, ranging from 30 to 40%, was observed in wet specimens even without LDH. However, with the addition of LDH, this trend changed, and the ultimate strength values became approximately equivalent for both dry and wet specimens. Despite this, the ultimate strength remained 20–30% lower compared to specimens without xylene and BYK.

**Figure 17:**
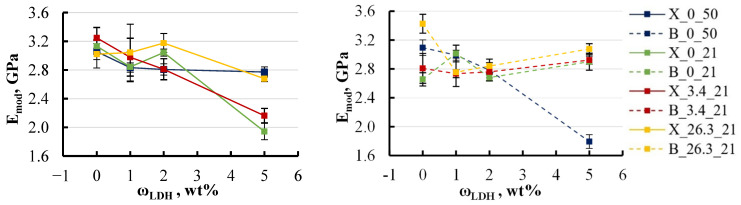
Effect of LDH content on the elastic modulus of soaked xylene (**left**) and BYK (**right**) specimens. Notations: X or B_salt content (wt%)_temperature (°C).

A closer examination of the effect of filler content on the elastic modulus revealed a trend toward decreasing modulus with increasing filler concentration in all water media (Figure 17).

For specimens containing xylene, the elastic modulus exhibited a reduction of 10–30% as the LDH content increased to 5 wt%, regardless of the type of aqueous environment. In contrast, specimens with BYK showed a more moderate reduction in elastic modulus, limited to around 10% under most conditions. However, a notable exception was observed when these specimens were exposed to hot water, where the modulus decreased by nearly 50%. This pronounced decrease suggests that the presence of BYK in specimens immersed in hot water significantly amplifies internal structural changes, resulting in a substantial modification of the material’s mechanical properties. This observation highlights the critical role that BYK plays as a wetting agent, potentially altering the interaction between moisture and the polymer matrix under elevated temperatures.

**Figure 18:**
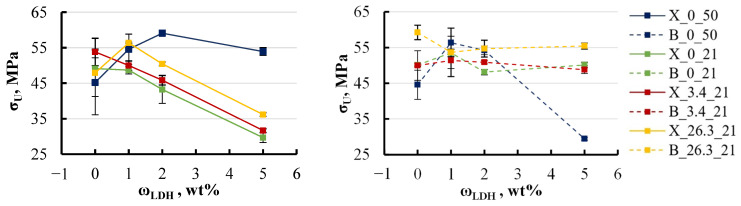
Effect of LDH content on the ultimate strength of soaked xylene (**left**) and BYK (**right**) specimens. Notations: X or B_salt content (wt%)_temperature (°C).

A similar pattern was noted for ultimate strength. In some groups, a slight increase in filler content to 2 wt% even led to an improvement in mechanical properties, but further increases in filler content caused a decline in these properties Figure 18.

For specimens containing xylene, the ultimate strength decreased by approximately 40% at an LDH content of 5 wt%, except for those exposed to hot water. Specimens in hot water initially displayed an ultimate strength that was 15% lower compared to those in other environments. However, as the LDH content increased, the ultimate strength exhibited a recovery, rising by 10–15%. In contrast, the behaviour of specimens with BYK was more stable. All groups, except for those subjected to hot water, showed minimal variations in ultimate strength, maintaining consistent values across different LDH concentrations. Nevertheless, as previously observed, specimens containing BYK that were exposed to hot water experienced a substantial reduction in ultimate strength. This underscores the significant impact of elevated temperatures on the mechanical properties of BYK-modified specimens, further highlighting the role of BYK in amplifying the effects of moisture under such conditions.

Focusing on the increase in temperature from 21 to 50 °C, the results indicate that both the elastic modulus and ultimate strength exhibited fluctuations within a range of 15% for specimens with LDH concentrations between 0 and 2 wt%. No clear trend of increase or decrease was observed in these properties at different temperatures. However, significant changes were noted in specimens containing 5 wt% of LDH. For xylene specimens, both the elastic modulus and ultimate strength increased by approximately one-third with the rise in temperature, whereas for BYK specimens, these values decreased by about one-third. These findings suggest that temperature has a limited effect on the mechanical properties of the material when the LDH content is 2 wt% or less.

**Figure 19:**
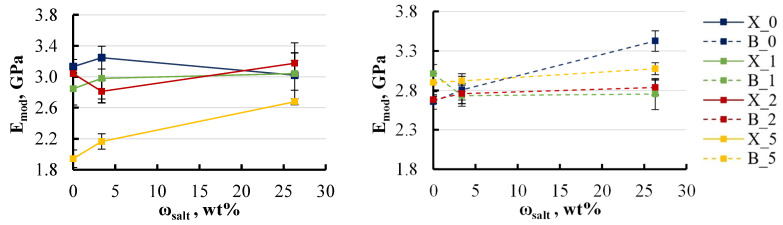
Effect of solution’s salt concentration on the elastic modulus of xylene (**left**) and BYK (**right**) specimens. Notations: X or B_LDH content (wt%).

The influence of salt concentration on the elastic modulus was not as dramatic, as shown in Figure 19.

**Figure 20:**
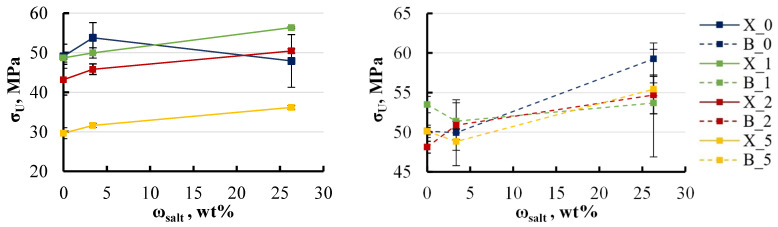
Effect of the solution’s salt concentration on the ultimate strength of xylene (**left**) and BYK (**right**) specimens. Notations: X or B_LDH content (wt%).

The effect of salt concentration on the elastic modulus of xylene specimens with an LDH content ranging from 0 to 2 wt% was minimal, resulting in fluctuations of just 5–8%. For xylene specimens containing 5 wt% LDH, the modulus was initially 40% lower in freshwater conditions but increased with higher salt concentrations in the surrounding medium. This behaviour can be explained by the reduction in equilibrium moisture content observed as salt concentration rises, as previously identified. The decrease in equilibrium moisture content implies that with higher salt concentrations, the specimens retain less moisture, thus reducing the impact of absorbed moisture on their mechanical properties. In contrast, BYK specimens demonstrated greater stability, which is consistent with earlier observations. The elastic modulus across all BYK-containing groups exhibited less variation, with changes limited to within 5%. The most notable increase in modulus, up to 15%, was observed in specimens without LDH when exposed to highly concentrated salt solutions. This suggests that BYK specimens maintain more consistent structural integrity under varying environmental conditions, making them less sensitive to changes in external moisture levels. Similar trends were observed in the behaviour of ultimate strength, as shown in Figure 20.

For all specimens, including both xylene and BYK ones, a slight increase in ultimate strength of 5–10% was observed as the salt concentration in the aqueous solution increased to 26 wt%. This increase is likely due to the reduced moisture content within the specimens when exposed to more concentrated salt solutions, leading to a diminished impact of moisture on the mechanical properties. An exception to this trend was noted in the xylene specimens without LDH. It is important to consider that these were the only specimens where xylene was introduced directly as a liquid rather than through a masterbatch. Thus, it is essential to consider the possibility that xylene may have a unique impact on the material’s properties. This influence could be particularly significant due to the direct addition of xylene, which may alter the internal structure of the specimens in a way that affects their mechanical behaviour differently compared to other formulations.

## 5. Validation

To validate the developed model for moisture concentration field calculation, several cases were examined. The first case involved a three-layer composite, where a reinforced plastic was coated on both sides with a layer of epoxy. For this scenario, each time step Δ*t* represented 10 h, with the surrounding humidity on both sides of the composite set to 100%. The sorption characteristics for each component and each layer within the composite are detailed in Table 2.

**Figure 21:**
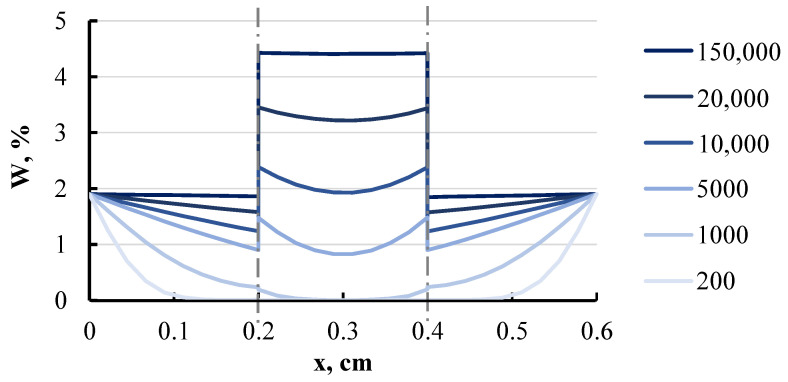
Modelled curves of moisture concentration in a three-layer plate at different moments: 200, 1000, 5000, 10,000, 20,000, and 150,000 h. The dashed line shows the connection between layers.

The calculation provided the concentration field within the composite over time. The simulated concentration curves for the three-layer composite are depicted in Figure 21.

At any given moment, the moisture content within layers remains uniform. Over a sufficiently long period, the layers reach saturation, as expected. At this point, the overall moisture content of the plate matches the equilibrium moisture content of the material under the specified relative humidity conditions, and the moisture potentials within the layers equalise with the surrounding relative humidity. This test serves as a critical validation of the accuracy of the algorithm and the computational program. This approach can also be extended to calculate the concentration in composites with a much larger number of layers and varying humidity levels on either side of the composite.

**Figure 22:**
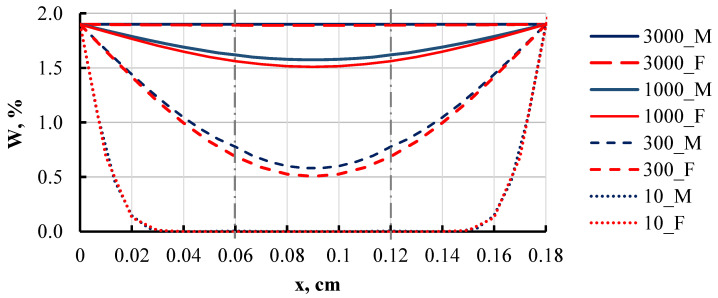
Modelled (M) and analytically solved (F) curves of moisture concentration in a three-layer film at different moments: 10, 300, 1000, and 3000 h. The dashed line shows the connection between layers.

To verify the model, the simulated data and curves were compared with analytically calculated curves using (3). For a simpler case, all three layers of the composite were taken from the same material, specifically epoxy resin. This allowed for a clear comparison of how well the model aligns with the analytical solution across all time intervals. The comparison of the analytical and simulated concentration curves is shown in Figure 22.

**Figure 23:**
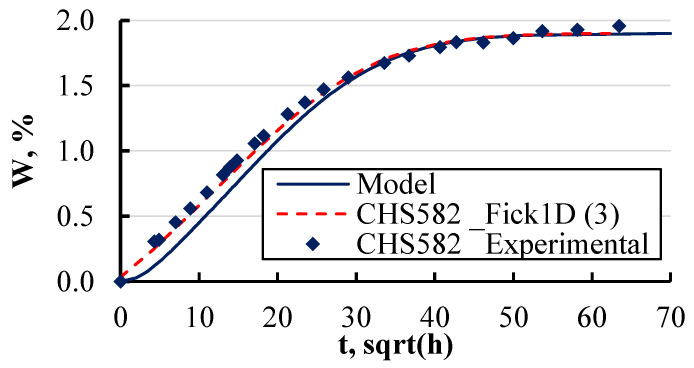
Experimental, modelled, and analytically solved sorption curves of a CHS 582 epoxy-based plate.

Additionally, the overall moisture content for the entire package was calculated. For comparison between the calculated and experimental data, the layer thickness in the calculations was adjusted to match the actual specimen thickness of 1 mm. As illustrated in Figure 23, the model’s predictions, analytical results, and experimental data are in close agreement, even as they approach the equilibrium moisture content. This confirms the accuracy of the model and its capability to predict moisture absorption in layers of varying thickness and materials.

The experimental data revealed that at elevated temperatures (Figure 10), the sorption curves become less predictable when using analytical methods. The developed numerical model exhibits comparable behaviour (Figure 23), indicating that it would also faces challenges in accurately predicting sorption at high temperatures. Consequently, it must be acknowledged that both analytical and numerical approaches have inherent limitations in predicting sorption curves under such conditions, leading to reduced accuracy in estimating moisture concentration. To enhance the reliability of calculations for sorption at elevated temperatures, a more detailed investigation into the nature of the sorption behaviour in such materials is required.

## 6. Conclusions

The research conducted in this study has provided significant insights into the behaviour of epoxy/LDH nanocomposites, particularly concerning their mechanical properties and moisture absorption characteristics under various environmental conditions. The key findings can be summarised as follows:

1. This study highlights that moisture absorption in epoxy/LDH nanocomposites is significantly influenced by environmental factors, such as temperature and salinity. Sorption curves indicate a gradual increase in equilibrium moisture content over time, which could be attributed to material degradation or leaching effects. Temperature was found to have a particularly strong impact, with the diffusion coefficient doubling when the temperature increased to 50 °C for most specimen groups. Additionally, an increase in salt concentration to 26 wt% led to a 50% reduction in equilibrium moisture content.
2. This study reveals a degradation in mechanical properties when epoxy/LDH nanocomposites are exposed to aqueous environments, especially at elevated temperatures. The elastic modulus decreased by approximately 5–8% with the addition of LDH up to 2 wt%, while the ultimate strength exhibited a more pronounced decline, dropping by 10–50% following immersion, particularly in hot water. Varying the LDH filler content from 0 to 5 wt% had minimal impact on the elastic modulus but did lead to a 5–10% reduction in ultimate strength. The study also examined the impact of base components, finding that xylene-modified specimens without LDH experienced a 10–15% reduction in ultimate strength. BYK-modified specimens showed greater stability.
3. The developed numerical model, designed to calculate moisture concentration fields in single- and multilayer materials, was rigorously validated against experimental and analytical data. The model effectively predicted moisture distribution over time, showing strong agreement with experimental observations. However, the model demonstrated limitations in predicting sorption curves at elevated temperatures, similarly to analytical methods. This validation process confirms the model’s reliability in simulating moisture absorption behaviour at near-room temperatures in complex, multilayered systems.

Future research should focus on optimising LDH incorporation methods to minimise agglomeration and improve the material outcome, potentially through alternative preparation techniques or modifying agents. Additionally, refining the numerical model to better predict sorption behaviour at elevated temperatures requires a deeper investigation into temperature-induced material changes. Further studies could also explore the impact of additional environmental stressors, such as UV exposure or cyclic loading, and assess the multifunctionality of LDH particles, particularly when loaded with active agents for enhanced anticorrosive or self-healing properties.

Overall, this study offers significant insights into the interactions between moisture absorption and mechanical degradation in epoxy/LDH nanocomposites. The findings underscore the role of environmental factors, such as temperature and salt concentration, in shaping the performance of these materials. Together with the validated model, this research provides a robust foundation for designing and optimising durable, environmentally resilient protective coatings.

## Figures and Tables

**Table 1:** Additives and their mass concentrations in 12 prepared compositions.

Method	Comp. Nr.	LDH %	Epoxy %	Hardener %	Xylene (X) %	BYK 180 (B) %
1	1	0	80.0	20.0	-	-
	2	0	68.0	17.0	15.0	-
	3	1	78.5	19.6	0.9	-
	4	2	76.9	19.2	1.9	-
	5	5	64.0	16.0	15.0	-
2	6	0	79.3	19.8	-	0.9
	7	1	78.9	19.7	-	0.4
	8	2	77.7	19.4	-	0.9
	9	5	74.3	18.6	-	2.2
3	10	1	79.2	19.8	-	-
	11	2	78.4	19.6	-	-
	12	5	76.0	19.0	-	-

**Table 2:** Sorption characteristics of components in the composite.

Material Name	Diffusion Coefficient	Layer Thickness	Density	Initial Relative Moisture Content	Sorption Isotherms		Number of Grid Points
					a^i^	b^i^	
	10^−6^, cm^2^/h	cm	g/cm^3^	%			n
CHS582	6.00	0.2	1.106	0.0	0.01900	1.00	10
Reinforced plastic	1.93	0.2	1.2	0.0	0.0471	1.51	10
CHS582	6.00	0.2	1.106	0.0	0.01900	1.00	10

## Data Availability

The original contributions presented in this study are included in the article. Further inquiries can be directed to the corresponding author.
